# Effect of *Elaeagnus Angustifolia* extract on in vitro wound healing of human dermal fibroblast cells

**DOI:** 10.1186/s13104-023-06644-0

**Published:** 2023-12-08

**Authors:** Ehsaneh Azaryan, Sepideh Sarfi, Seyede Fatemeh Hosseini, Mansoore Saharkhiz, Khadijeh vazifeshenas-Darmiyan, Mohsen Naseri

**Affiliations:** 1 Cellular and Molecular Research Center, Department of Molecular Medicine, Birjand University of Medical Sciences, Birjand, Iran; 2 Student Research Committee, Birjand University of Medical Sciences, Birjand, Iran; 3 Department of Immunology, Faculty of Medicine, Birjand University of Medical Sciences, Birjand, Iran; 4 Department of Anatomy, Tabas School of Nursing, Birjand University of Medical sciences, Birjand, Iran; 5 PhD student in Clinical Biochemistry, Faculty of Medicine, Shahid Sadoughi University of Medical Sciences, Yazd, Iran; 6 Cellular and Molecular Research Center, Birjand University of Medical Sciences, Birjand, Iran

**Keywords:** *Elaeagnus Angustifolia*, Human dermal fibroblast, Wound healing

## Abstract

**Aim:**

The purpose of this study was to determine the impact of *Elaeagnus Angustifolia* extract (EA) on human dermal fibroblast (HDF) survival, migration, and wound healing-related genes.

**Methods:**

After preparing the hydroalcoholic extract of EA, MTT and scratch tests were used to determine the effect of EA on the viability and migration of HDFs. In addition, the quantitative polymerase chain reaction (q-PCR) was conducted to evaluate the impact of EA on the expression of wound healing-related genes in HDFs.

**Result:**

According to the MTT test, a nontoxic concentration of EA (100 µg/ml) was obtained for further investigations. The scratch test results demonstrated that EA improved HDFs’ capacity to migrate when compared to the control group. Additionally, q-PCR results revealed that EA could significantly increase wound healing-related genes (VEGF-A, HLA-G5, and IL-6) in comparison with the control group.

**Conclusions:**

The EA could have a significant impact on the viability and migration of HDFs. Also, EA increased the expression of wound healing-related genes.

## Introduction

The skin acts as a multilayer interface between the body and the outside world, controlling temperature, avoiding dehydration, preventing infection, and transporting water. When the skin is damaged, the body immediately begins the recovery process. To repair skin tissue injuries, a well-coordinated interaction of proliferation and migration of cells, collagen formation, wound remodeling, and angiogenesis is necessary. A serious global public health problem that can be fatal is impaired skin wound healing. Despite several therapy attempts to encourage wound healing, the best treatment strategies are still being developed [1]. Thus, several laboratory investigations and clinical trials have been carried out to look into novel methods of promoting wound healing by the use of either contemporary physical and pharmacological therapies or phytotherapy [2].

Medicinal plants have been used to cure diseases for thousands of years. *Elaeagnus Angustifolia* (EA) is a plant that is extensively utilized in Iranian herbal medicine [3]. Secondary metabolites, nutrients, minerals, amino acids, and polysaccharides are all found in the EA [4]. Conventional medicine manipulates EA extracts as a muscle relaxant, antipyretic, antinociceptive, and anti-inflammatory remedy [5–7]. According to research, the EA contains a variety of chemical substances such as amino acids, flavonoids, phenolic compounds, polysaccharides, and other vital nutrients [8]. Flavonoids are recognized for their antibacterial properties, which aid in wound healing and epidermal regeneration in the skin [9].

In another research, it has been shown that the extract of EA fruit would alleviate pain and inflammation, likewise expediting wound healing [10]. The success of this plant in wound healing might be attributed to an increase in re-epithelization and collagen deposition in the wound following applications of fruit extract [9].

Fibroblasts are distinguished from other cells like epithelial and endothelial cells by their distinct spindle-shaped appearance [11]. These cells have been employed in clinical investigations, mostly for wound healing therapies [12]. Fibroblasts, in particular, have emerged as crucial immunological monitoring cells. When they identify pathogenic stimuli as well as injury and pathogen-associated molecular patterns, they stimulate and regulate the immune response [11]. Essential polysaccharides with antiradiation, immunomodulatory, and antioxidant activities are present in EA. Significant immunological properties exist in the polysaccharides from *Elaeagnus angustifolia L*. (EAP). They might considerably raise the spleen and thymus indices, boost NK cell activity, encourage peritoneal phagocytosis, and enhance blood levels of IL-2, IFN-γ and IgG [8, 13]. In fact, studies on EA now focus mostly on its phytochemical components and nutritional and therapeutic benefits, and research on its effects on wound healing is still rare. The current study aimed to assess fibroblast wound repair/regeneration skills following EA therapy, with the notion that EA might effectively optimize these cells. Therefore, we evaluate viability, migration, as well as wound healing related genes in EA-treated human dermal fibroblasts (HDFs).

## Materials and methods

### EA hydroalcoholic extract preparation

In previous studies, we explored the preparation of EA hydroalcoholic extract [14, 15]. The fruits of EA were collected from South Khorasan (Birjand) region and identified and approved by Dr. Sayyedeh Fatemeh Askari as *Elaeagnus Angustifolia L*. A specimen was kept in the Herbarium Center of the Faculty of Pharmacy at Birjand University of Medical Sciences (Voucher number 221). In short, the solvents for the maceration technique of extracting 40 g of EA powder were 320 ml of methanol and 80 ml of distilled water. The extracted solution was then filtered and concentrated using a rotating vacuum, and the extract was stored at 4 °C.

### MTT assay

In this study, HDFs from the Pasteur Institute cell bank in Iran (NCBI Code: C646) were used. Cells were cultured in DMEM culture media (Gibco) enriched with 10% fetal bovine serum (FBS; Sigma) and 1% penicillin and streptomycin (pen/strep; Sigma) at 37 °C and 5% CO2. The cytotoxicity of EA on HDFs was determined using the MTT test. The cells were cultivated on a 96-well plate with 10^4^ cells per well for 24 h at 37 °C and 5% CO2 until they achieved a confluence of 70–80%. The cells were then exposed to various concentrations of EA (0, 5, 10, 25, 50, 100, 250, and 500 µg/ml) and incubated for another 24 h. Finally, each well received 20 µl of MTT, and the plate was incubated for 4 hours in the dark at 37 °C, the media was changed with 100 µL dimethyl sulfoxide (DMSO; Sigma). A microplate reader was used to measure absorbance at 570 and 630 nm (Biotek Epoch, Winooski, VT).

### Wound scratch assay

HDFs were cultured on 12-well plates (SPL, Korea) with 2 × 10^5^ cells per well. A scratch wound was achieved on monolayer culture in each well by a 200 µl pipette tip. After removing the media, the cells were washed twice with PBS to eliminate the unattached cells. They were instantly treated with a 100 µg/ml concentration of EA. Using the inverted light microscope, the scratch areas of each well were evaluated. The images were obtained at ×40 magnification at 0, 12 and 24 h after scratching, and the rate of wound closure was calculated using Image J software.

### Real-time PCR analysis

The total RNA was obtained using the kit process (Pars Tous, Tehran, Iran), and the extracted RNA quality was evaluated using a nanodrop (Biotek Epoch). For cDNA synthesis, a Pars Tous kit was employed. qPCR was performed using primers (Table 1) designed for the human leukocyte antigen-G5 (HLA-G5), Interleukin-6 (IL-6), and vascular endothelial growth factor (VEGF-A) genes on an Applied Biosystems Step One Plus real-time PCR machine using Quanti Tect SYBR Green qPCR Master Mix. The quantities critical threshold (Ct) of the target gene were normalized with the quantities of the internal control (GAPDH).

**Table 1:** Sequences of primers used for real-time PCR

Name	Forward	Reverse
VEGF-A	AGGGCAGAATCATCACGAAGT	AGGGTCTCGATTGGATGGCA
HLA-G5	CTGAGATGGAAGCAGTCTT	GCTCCCTCCTTTTCAATCT
IL-6	AGACTTGCCTGGTGAAAATCA	GCTCTGGCTTGTTCCTCACT
GAPDH	CGAACCTCTCTGCTCCTCCTGTTCG	CATGGTGTCTGAGCGATGTGG

### Statistical analysis

All data were shown in mean ± SD and each experiment was carried out in duplicate (n = 2). The difference between the groups was analyzed with GraphPad Prism 9 (GraphPad Software, Inc, La Jolla, CA). One-way analysis of variance (ANOVA) followed by Tukey’s multiple comparison test was used to analysis of MTT and scratch test, and Student’s t-test was used to evaluate the results of gene expression. Statistical significance level was p < 0.05.

## Results

### Effect of EA on fibroblast viability

**Fig. 1:**
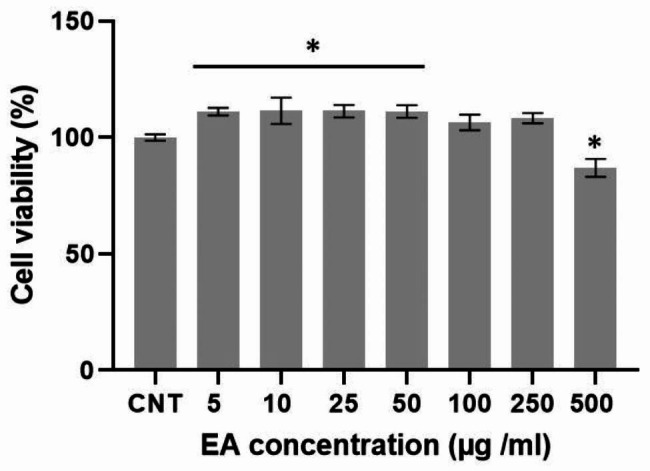
Viability assay for EA-treated HDFs on 24 h. Significant differences compared to control were indicated: *p < 0.05; CNT, Control

The fibroblasts showed significantly higher cell viability when treated with EA at doses less than or equal to 50 µg/ml when compared to the control group (p < 0.05). while there was no statistically significant difference in cell toxicity or proliferation at 100 and 250 µg/ml concentration compared to the control group (p > 0.05). Moreover, at 500 µg/ml, the viability of the cells significantly declined (p < 0.05). The nontoxic concentration of EA (100 µg/ml) was utilized for the investigations that followed (Fig. 1).

### Effect of EA on fibroblast migration

**Fig. 2:**
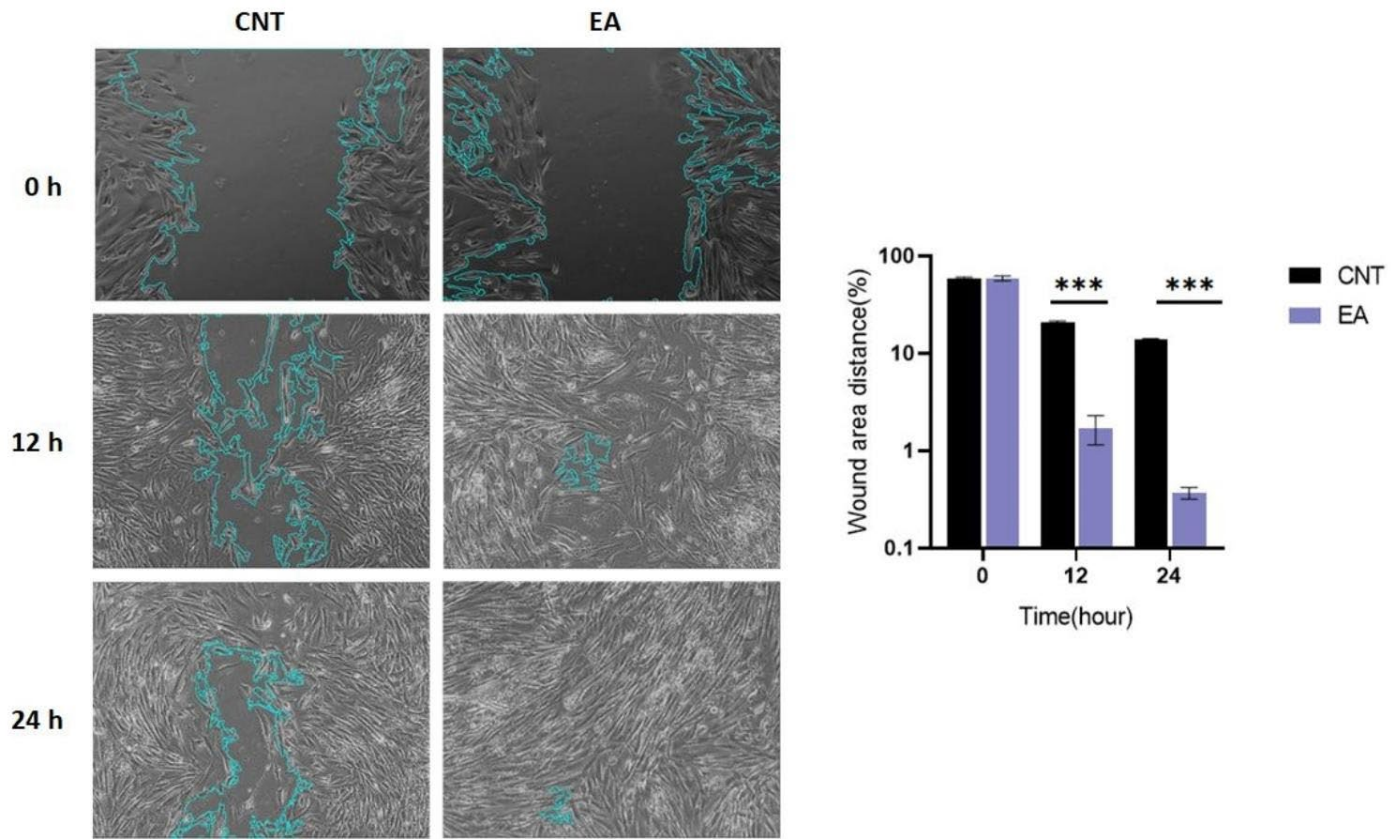
Quantitative analysis of the migration area. Representative images are presented after 12 and 24 h of HDFs treatment with EA cells in a wound scratch assay. Significant differences compared to control were indicated: ***p < 0.001; CNT, Control

HDFs were exposed to the EA (100 µg/ml), and the scratch test was used to determine any alterations in the cells’ capacity to migrate. Results showed that EA improved fibroblast cell migration rate compared to control after 12 h of treatment (p < 0.001) (Fig. 2). The wound healed entirely after 24 h at a concentration of 100 µg/ml, however, the control group did not heal completely.

### Effect of EA on mRNA expression of IL-6, VEGF-A and HLA-G5

**Fig. 3:**
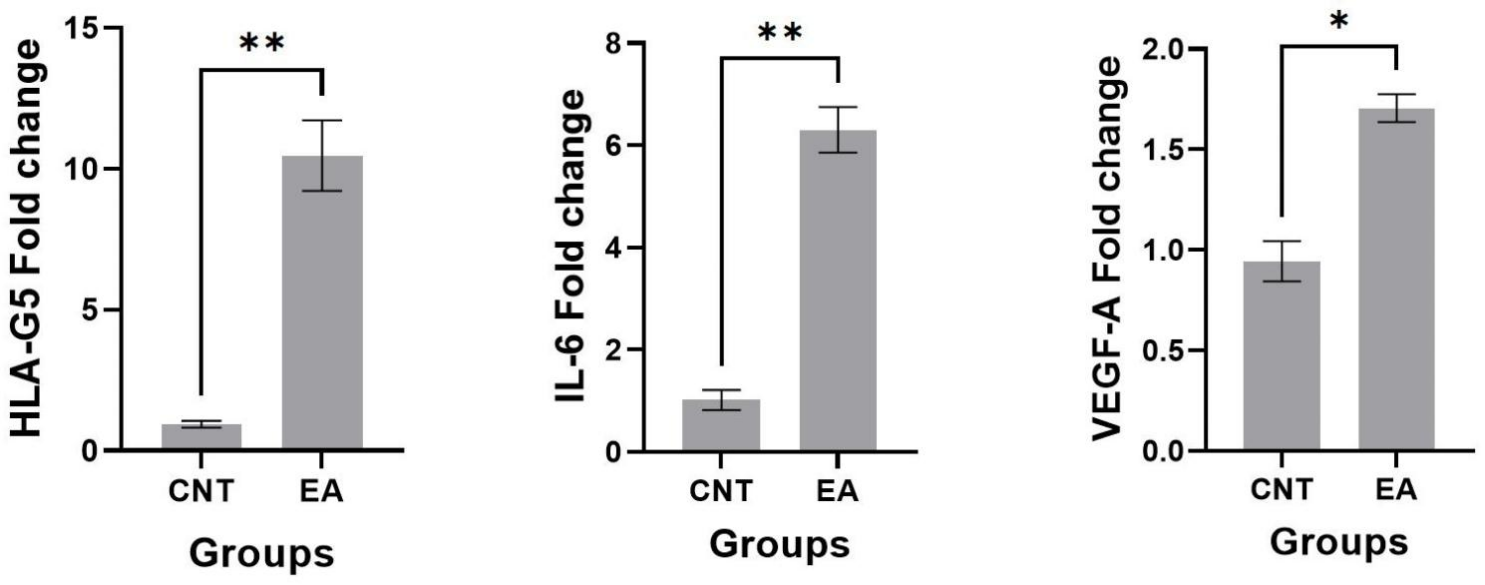
Comparison of the gene’s expression levels: HLA-G5, IL-6, and VEGF-A. *p < 0.05, **p < 0.01 for each group compared to the control group; CNT, Control

Relative expression of HLA-G5, IL-6, and VEGF-A genes were determined in the cells cultured in complete medium with or without EA extract at 24 h. The real-time PCR analysis showed (Fig. 3) that treatment of cells with EA extract significantly increased the expression of the HLA-G5, IL-6, and VEGF-A genes compared to the control group. (P < 0.01), (P < 0.01) and (P < 0.05) respectively.

## Discussion

The goal of this study was to give early data on the effects of EA extract on HDF with the expectation that the results of previous investigations, as well as future studies, may open up new avenues for optimizing the therapeutic effectiveness of fibroblasts. As a result, wound healing-related biomarker expression in EA extract-treated HDFs was investigated in this work on the hypothesis that this plant extract can effectively induce them. Therefore, in this study, the effects of EA on the viability, and migration of HDF cells were assessed.

Although several chemical components of EA may have wound-healing activities, the current study attempted to investigate the effects of each of them as a complete compound rather than a particular ingredient. According to phytochemical investigations, the aqueous fruit extract of EA includes flavonoids, sitosterols, cardiac glycosides, terpenoids, vitamins B and A, and vitamin K [10]. Vitamin A has a variety of functions in wound healing, including antioxidant activity, increased fibroblast proliferation, modulation of cellular differentiation and proliferation, increased collagen deposition, and hyaluronate production [16].

Natanzi and colleagues revealed that EA aqueous extract enhanced cutaneous wound healing, which may be attributed to accelerated re-epithelialization and collagen deposition in the wound, and so it may be regarded as a therapeutic factor for wound healing [10].

Our findings showed that at optimal EA concentration (100 µg/ml), HDF cells moved significantly and totally repaired the wound within 24 h (Fig. 2).

Accelerating angiogenesis at wound sites is crucial for effective wound healing. VEGF is a potent pro-angiogenic molecule that is suitable for the therapeutic activation of blood vessel expansion [17]. VEGF enhances collagen synthesis and epithelialization in addition to promoting angiogenesis, which speeds up the healing of wounds [18]. Furthermore, employing a pig model revealed that the 3D printed Gel MA-VEGF hydrogel aided wound healing by encouraging collagen deposition and angiogenesis at the wound area [19].

VEGF-A and IL-6 expressions are crucial to the mechanisms involved in wound healing. IL-6 can stimulate angiogenesis by increasing the production of VEGF [20]. Furthermore, Lin et al. demonstrated that VEGF gene expression was decreased in the wound area of IL-6 knockout mice in comparison with wild-type mice [21]. IL-6 and VEGF expressions perform an essential function in the wound repair process. VEGF serves as an endothelial cell stimulator, chemotactic molecule, and activator of vascular permeability, whereas IL-6 regulates immune responses, and is crucial for rapid wound healing [20].

There is conclusive evidence that IL-6 regulates leukocyte infiltration, angiogenesis, and collagen deposition throughout the wound-healing process [22].

In line with these studies, our results also depicted high expression of these genes in EA-treated HDF. This demonstrates that there may be a connection between the VEGF and IL-6 upregulation effects of EA.

HLA-G5 is a key immunological tolerance factor in the human body, and its expression is essential for immunomodulatory purposes [15, 23]. Also, this molecule may serve as a biomarker for the accurate identification of MSCs with significant immunomodulatory capabilities [24]. It has been noted that the morphology, gene expression profiles, surface markers, proliferation, differentiation, and immunomodulatory abilities of fibroblasts and mesenchymal stromal cells are comparable [12]. As a result, we evaluated the expression of this gene in HDF for the first time and demonstrated that EA could increase it. Immunomodulatory properties not only affect immune cell responses, but also boost the activity of other cells, such as human keratinocytes, fibroblasts, and endothelial cells [25]. According to our findings, EA extract may aid in speeding up the wound closure process in this way. Our findings revealed that EA extract had the effective wound healing characteristics and might be used as a therapeutic agent for chronic wounds and their consequences.

## Conclusion

**Fig. 4:**
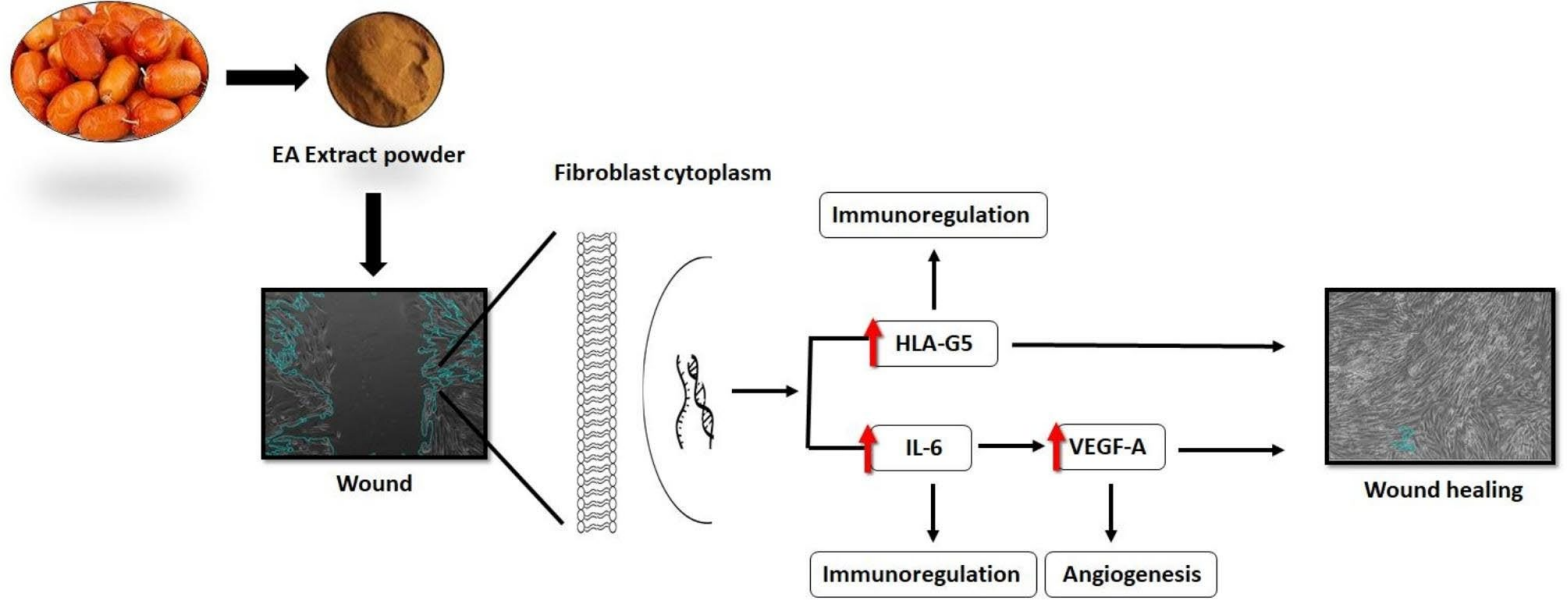
EA increased the expression of wound healing-related genes in HDFs.

Fibroblasts are the potential cells of the dermis participating in wound healing via migration and proliferation. In this study, EA had a significant impact on the viability and migration of HDFs. Also, this investigation offered early insights into the effects of EA on altering the expression of the markers associated with wound healing on fibroblasts and may be helpful in expanding the cells’ capacity to heal wounds (Fig. 4). Because of this, the results lay the groundwork for future investigations into the biological consequences of EA on fibroblasts, which might help with the progression of cell-based therapeutics.

### Limitations

Despite the fact that our findings are encouraging, further investigation using in vivo models will be necessary in the future to demonstrate EA’s efficacy as an inducer of wound healing.

## Data Availability

All data of the study are available from the corresponding author upon reasonable request.

## Abbreviations

EA: Elaeagnus Angustifolia
HDF: Human dermal fibroblast
q-PCR: Quantitative polymerase chain reaction
VEGF: Vascular endothelial growth factor
HLA-G5: Human leukocyte antigen-G5
IL-6: Interleukin-6
